# Fatal Liver and Lung Alveolar Echinococcosis with Newly Developed Neurologic Symptoms due to the Brain Involvement

**DOI:** 10.1055/s-0036-1592122

**Published:** 2016-09-04

**Authors:** Robertas Kvascevicius, Ona Lapteva, Omar Al Awar, Egle Audronyte, Laura Neverauskiene, Eleonora Kvasceviciene, Vitalijus Sokolovas, Kestutis Strupas, Audrone Marcinkute, Peter Deplazes, Beat Müllhaupt

**Affiliations:** 1 Centre of Neurosurgery, Vilnius University Hospital Santariskiu Klinikos, Vilnius, Lithuania; 2 Centre of Neurology, Vilnius University Hospital Santariskiu Klinikos, Vilnius, Lithuania; 3 Department of Pathology, Republican Vilnius University Hospital, Vilnius, Lithuania; 4 Department of Radiology, Republican Vilnius University Hospital, Vilnius, Lithuania; 5 Centre of Abdominal Surgery, Vilnius University Hospital Santariskiu Klinikos, Vilnius, Lithuania; 6 Hospital of Infectious Diseases and Tuberculosis, Affiliate of Vilnius University Hospital Santariskiu Klinikos, Vilnius, Lithuania; 7 Institute of Parasitology, University of Zürich, Zürich, Switzerland; 8 Swiss HBP Center, University Hospital of Zürich, Zürich, Switzerland

**Keywords:** alveolar echinococcosis, *Echinococcus multilocularis*, cerebral alveolar echinococcosis

## Abstract

The fox tapeworm *Echinococcus multilocularis* causes human alveolar echinococcosis, commonly affecting the liver. However, in ∼1% of cases, systematic spread of the disease involves the brain as well. A patient had a 6-year history of liver and lung alveolar echinococcosis that was considered not suitable for surgery, and treatment with albendazole was introduced. After the appearance of neurologic disturbances, an intracranial mass lesion was demonstrated by radiologic imaging. The lesion was surgically removed, and histologic analysis revealed metacestode tissue of *E. multilocularis* . Despite the surgical resection of the lesion, the patient died of progression of systemic alveolar echinococcosis. The authors highly recommend implementing neurologic monitoring to the follow-up algorithm for patients with systemically disseminated alveolar echinococcosis. When neurologic symptoms occur, radiologic imaging of the brain should be obtained immediately. Surgery should be considered for all intracranial echinococcal lesions, unless the lesion is located in the eloquent brain area.

## Case Report

**Fig. 1:**
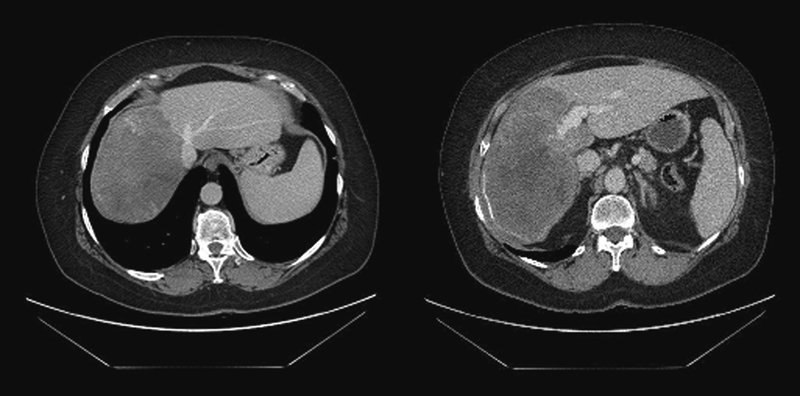
Abdominal computed tomography with contrast media. (Left) A mass with diffuse amorphous calcification in the right liver lobe. (Right) The lesion with calcified margins occupying the right liver lobe and involving the portal vein system.

**Fig. 2:**
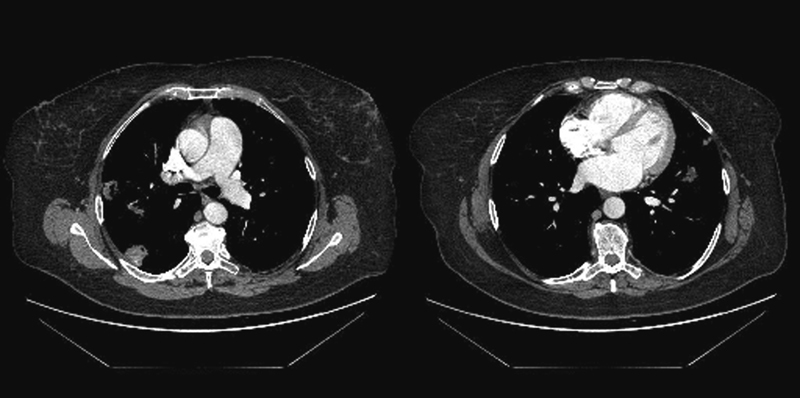
Thoracic computed tomography with contrast media. (Left) The calcified lesions with lobulated contours in the right lobe of lungs. (Right) The lesion in the left lobe of lungs.

A 63-year-old woman residing in northern Lithuania was admitted to the Department of Neurosurgery at Republican Vilnius University Hospital with severe headache, progressive left-sided hemiparesis, and seizures. These symptoms, along with ataxia and left-sided facial nerve palsy, were present for several weeks and gradually worsened. The patient was diagnosed 6 years before with liver alveolar echinococcosis (AE) stage IV P4N4M1 with transdiaphragmatic bilateral pulmonary dissemination ( Figs. 1 , 2 ). 1 The case was considered nonsurgical, and treatment with albendazole 400 mg twice a day was initiated. This treatment resulted in elevation of hepatic enzymes, and the dose was reduced to 200 mg per day. The patient developed pruritus, headache, and cardiac pain. At the same year prior to the admission for the brain AE lesion treatment, the patient was treated for obstructive jaundice at the Department of Abdominal Surgery at Vilnius University Hospital Santariškių Klinikos. An endoscopic retrograde cholangiography and papillotomy with stent insertion were performed. By all measures, the patient had a terminal and further progressing systematic disease.

**Fig. 3:**
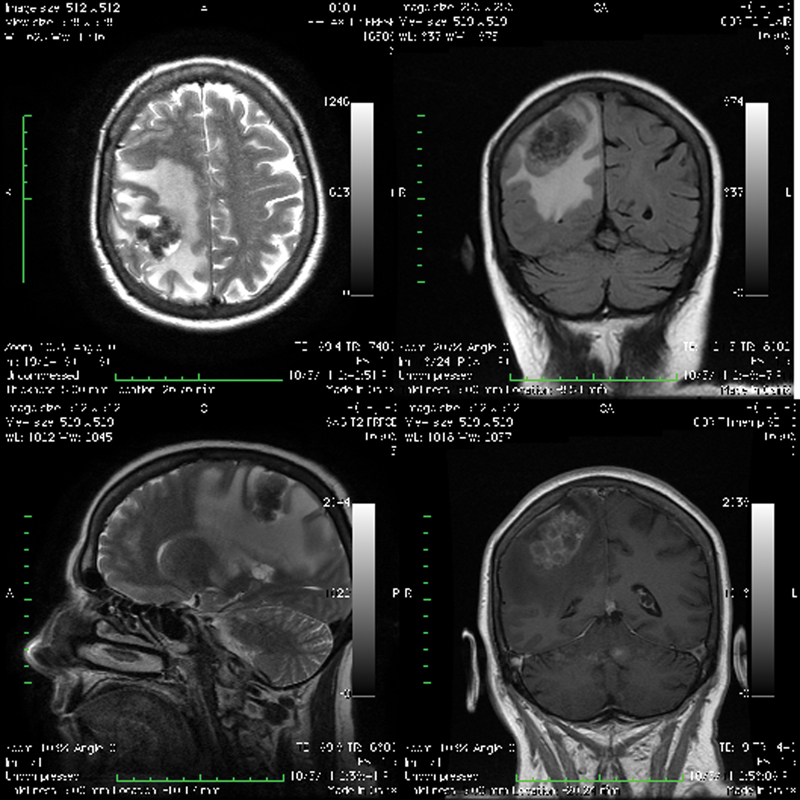
Brain magnetic resonance imaging. Axial view T2-weighted image (upper left) and coronal view T2-weighted image (bottom left) showing the irregular hypo-/hyperintense nodular lesion in the right postcentral gyrus with an extensive perifocal edema. Coronal fluid-attenuated inversion recovery image (upper right) showing the nodular iso-/hypointense lesion with a massive perifocal edema in the right parietal lobe. Coronal T1-weighted image with contrast enhancement (bottom right) showing the multilocular cystic lesion with a perifocal edema resembling a bunch of grapes.

Magnetic resonance imaging (MRI) of the brain demonstrated an irregular, contrast-enhancing, 40 × 34 × 25-mm nodular lesion in the postcentral gyrus surrounded by extensive perifocal edema ( Fig. 3 ).

**Fig. 4:**
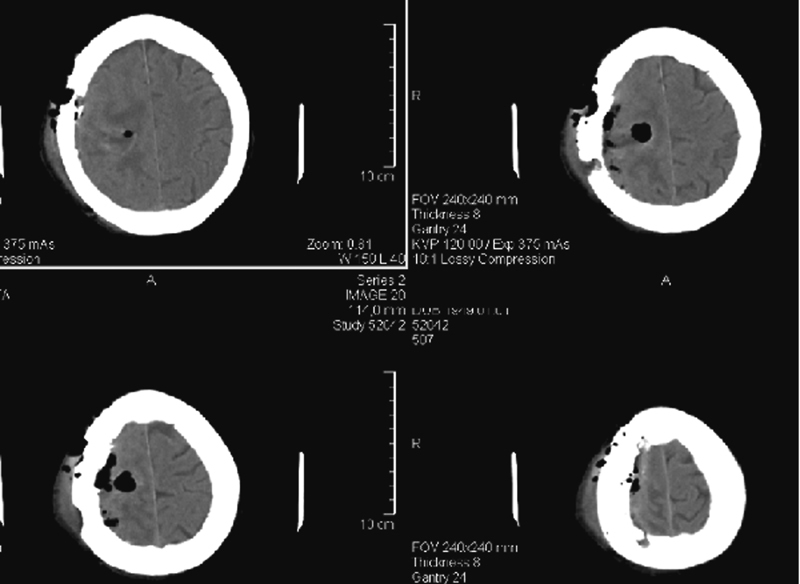
Postoperative computed tomography. This image shows the radical removal of the lesion.

During surgery, a solid lesion of the right motor cortex was identified and totally removed. The lesion was white to gray, multilocular, limited, and easily distinguishable from the normal brain tissue ( Fig. 4 ).

**Fig. 5:**
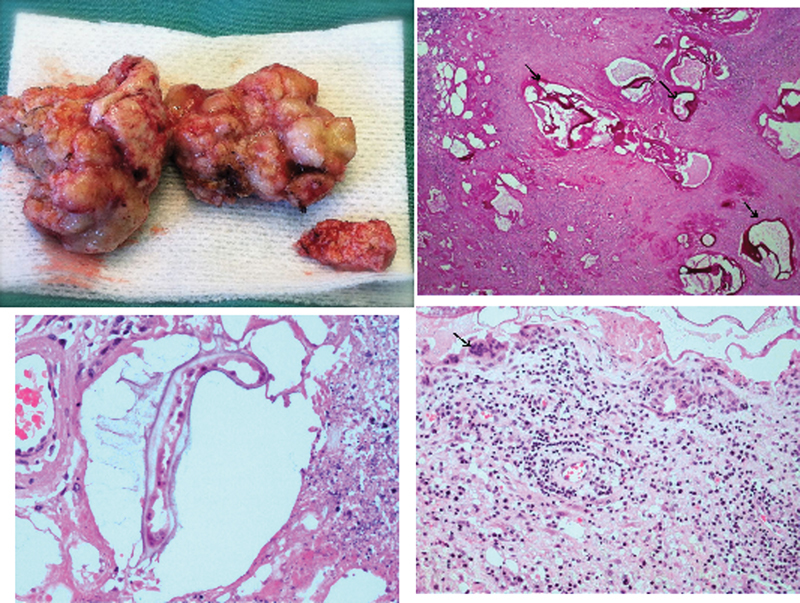
(Upper left) The gross pathologic specimen of the lesion removed during surgery. (Upper right) The section shows periodic acid-Schiff (PAS)-positive cuticle layer characteristic of *Echinococcus multilocularis* cysts (arrows). PAS stain. (Lower left) The cystic lesion with parasite surrounded by necrotic tissue. Hematoxylin and eosin stain. (Lower right) The surrounding brain tissue infiltrated by lymphocytes, plasmocytes, and multinucleated giant cells (arrow).

The histopathologic examination of the resected lesion revealed multiple cysts with periodic acid-Schiff-positive cuticular layers surrounded by necrosis, heavy infiltration of inflammatory cells, and granulomatous reaction characterized by epithelioid cells and multinucleated giant cells. These morphologic findings confirmed the suspected diagnosis of AE ( Fig. 5 ).

The patient had a full neurologic recovery postoperatively. On the second postoperative day, she experienced abdominal pain. Clinical and radiologic investigation (plain abdominal X-ray, abdominal ultrasound) demonstrated no signs of peritonitis or intestinal obstruction (ileus), but ascites was noted, which increased progressively to produce respiration insufficiency. On the fifth postoperative day, the patient died of cardiopulmonary insufficiency.

## Discussion

In Lithuania, AE caused by the fox tapeworm *Echinococcus multilocularis* is of increasing public health concern, with the highest incidences recorded in Europe, varying from 0.03 per 100,000 inhabitants in 2004 to 0.57 in 2009 and 0.74 in 2012. AE primarily affects the liver but in a quarter of the cases in Lithuania extrahepatic disseminations were observed, and in 35.4% of the cases, the survival was less than 1 year from diagnosis due to late diagnosis at stage IV of the disease. For the control of this zoonosis, timely diagnosis and treatment in humans as well as the development of local control programs present major challenges.

AE with resultant brain involvement is rare (1%). 2 3 4 5 6 Brain involvement generally presents with focal neurologic deficit and features of raised intracranial pressure. 5 The symptoms of raised intracranial pressure are headache, nausea, and vomiting. Other neurologic disturbances may include aphasia, dysarthria, seizures, hemiparesis, vision impairment, and cranial nerves palsies. 2 3 6 7 8 9

Cerebral lesions can be solitary and multifocal. In general, they are supratentorial and distributed within the middle cerebral artery. 4 10 In cranial computed tomography and MRI, they appear as solid, semisolid, or multilocular cystic lesions resembling a bunch of grapes. Calcification and perifocal edema are common. The lesions show contrast enhancement. Ringlike, heterogeneous, nodular, and cauliflower-like enhancement patterns have been reported. 3 4 5 6 8 10 11 12 It is important to differentiate AE from cerebral malignancies (metastasis, high-grade gliomas) and other infective cerebral diseases like tuberculosis, bacterial abscess, fungal infection, toxoplasmosis, cysticercosis, and cystic echinococcosis. 4 6

Currently, there is no consensus on treatment of brain AE. Radical resection is the treatment of choice, except for the cases when a surgical approach may cause mortality or considerable morbidity (i.e., when the lesion is located in eloquent brain areas). The patients undergo chemotherapy with benzimidazole derivates (albendazole, mebendazole) for 2 years after the radical surgery. Albendazole is a first-choice drug for the treatment of cerebral AE, because it penetrates the blood–brain barrier better than mebendazole. If resection was incomplete or if the case is nonsurgical, long-term or even lifelong treatment with albendazole is initiated. 6 9 13 14 Gamma knife radiosurgery may also be considered. 15

We report our experience of surgical treatment of brain AE. Although extremely rare (<1%), cerebral echinococcosis should be considered as a differential diagnosis of intracranial mass lesions. In general, clinicians must be well aware of the echinococcosis problem and must carefully consider the clinical (i.e., medical history of echinococcosis) and epidemiologic details (i.e., direct contact with the definitive hosts, residence in an *Echinococcus* -endemic region) when examining the patient. The appropriate diagnostic tools help to establish the correct diagnosis quickly. Specific medical therapy prevents the progression and systemic spread of echinococcosis.

The AE lesion in the brain may have been a consequence of metastatic spread of the disease from the primary lesion in the liver, which could have happened during a time of inappropriate medical control of the disease. Such a moment might have occurred when the patient experienced adverse effects from the medical treatment, which could have led to the patient taking the drugs irregularly or in inadequate doses.

Another explanation may be that the multiple oncospheres' invasion resulted in all three primary lesions in the liver, lung, and brain, but for some reasons the brain lesion became clinically evident the latest. Aoki et al reported a case of a patient infected with at least two oncospheres that developed into two small primary separate cysts in different liver segments. 16

Our patient had her first brain MRI scan when neurologic symptoms appeared and not at the time of diagnosis of the disease, so the primary brain lesion/metastatic brain lesion cannot be proved or denied.

If we presume that the brain lesion was a primary lesion along with the liver and lung lesions, why then was it clinically silent for more than 13 years? Is it possible that liver echinococcosis progressed while the brain lesion was occult despite the poor drug penetration into the brain tissue and fewer intracranial mass compensatory mechanisms? This question could only be answered if neurologic and neuroradiologic investigations had been initiated at the time of diagnosis of disseminated AE.

Difficulties in controlling the dangerous progression of AE led to the death of the patient. At the beginning, the patient was diagnosed as being in the terminal stage of the disease. We believe that the asymptomatic period may have lasted for many years. Multidisciplinary screening and follow-up along with the albendazole therapy and palliative surgery increased the survival for 7 years after the establishment of the diagnosis.

## Conclusions

The authors highly recommend introducing neurologic screening into the treatment and follow-up algorithm for the patients with disseminated AE even though no neurologic disturbances are evident. This recommendation is especially important when the patient has the disease for a long time or does not get adequate treatment. When neurologic symptoms occur, brain MRI scan should be obtained immediately. Surgery should be considered for all intracranial echinococcocal lesions, unless the lesion is located in the eloquent brain area. In summary, clinicians must be aware of this condition to allow early diagnosis, facilitate referral, and ensure prompt treatment. Proper medical treatment, regular long-term multidisciplinary follow-up, and early surgical interventions may improve the clinical outcome and survival.
